# Beneficial effects of the first case of washed microbiota transplantation for postorgasmic illness syndrome: a case report

**DOI:** 10.1093/sexmed/qfae015

**Published:** 2024-03-27

**Authors:** Yong-Xi Quan, Ye-Dong Lao, Hui-Yi Wu, Xing-Xiang He, Li-Hao Wu

**Affiliations:** Department of Gastroenterology, Research Center for Engineering Techniques of Microbiota-Targeted Therapies of Guangdong Province, The First Affiliated Hospital of Guangdong Pharmaceutical University, Guangzhou 510080, China; Department of Gastroenterology, Research Center for Engineering Techniques of Microbiota-Targeted Therapies of Guangdong Province, The First Affiliated Hospital of Guangdong Pharmaceutical University, Guangzhou 510080, China; Department of Gastroenterology, Research Center for Engineering Techniques of Microbiota-Targeted Therapies of Guangdong Province, The First Affiliated Hospital of Guangdong Pharmaceutical University, Guangzhou 510080, China; Department of Gastroenterology, Research Center for Engineering Techniques of Microbiota-Targeted Therapies of Guangdong Province, The First Affiliated Hospital of Guangdong Pharmaceutical University, Guangzhou 510080, China; Department of Gastroenterology, Research Center for Engineering Techniques of Microbiota-Targeted Therapies of Guangdong Province, The First Affiliated Hospital of Guangdong Pharmaceutical University, Guangzhou 510080, China

**Keywords:** postorgasmic illness syndrome, washed microbiota transplantation, fecal microbiota transplantation, sexual dysfunction, microbiota

## Abstract

**Introduction:**

Postorgasmic illness syndrome (POIS) is characterized by allergic symptoms and flu-like illness after ejaculation. There are still no effective treatments for POIS.

**Aim:**

To report the first case of washed microbiota transplantation (WMT) to treat patient with POIS.

**Methods:**

Data were collected from a patient with POIS who had received 3 courses of WMT: self-rating scale of POIS symptoms, Self-rating Anxiety Scale, Self-rating Depression Scale, and Symptom Checklist 90. The patient’s stool samples for 16sDNA sequencing were collected 1 month after WMT.

**Results:**

POIS symptoms improved after WMT. Scores decreased from baseline after WMT: self-rating scale of POIS symptoms (before WMT, 16; after first, 16; after second, 8; after third, 9), Self-rating Anxiety Scale (45, 42.5, 37.5, 45), Self-rating Depression Scale (63.75, 58.75, 47.5, 50), and Symptom Checklist 90 (143, 140, 109, 149). Characteristics of the patient’s gut microbiota changed. At the genus level, the relative abundance of beneficial bacteria increased, and some opportunistic pathogenic bacteria decreased.

**Conclusion:**

WMT may be an effective and safe choice for the treatment of patients with POIS by changing the gut microbiota of the host.

## Introduction

Postorgasmic illness syndrome (POIS) was first proposed by Waldinger and Schweitzer in 2 case reports in 2002.^1^ POIS, a syndrome in men, occurs seconds or minutes after ejaculation, which mainly consists of allergic symptoms and flu-like illness, such as fever, perspiration, chills, pain muscles, itching eyes, congestion, runny nose, and sneezing. These symptoms can last 4 to 7 days. Recent literature has reported the following as treatment: benzodiazepines, selective serotonin reuptake inhibitors, hyposensitization treatment, intralymphatic immunotherapy, subcutaneous injections of human chorionic gonadotropin, combination of testosterone enanthate and nonsteroidal anti-inflammatory drugs, highly selective alpha 1A blocker, and surgery for bilateral epididymectomy and vasoligation .^2^ However, there is no uniform standard or consensus for the treatment of POIS because of the lack of extensive clinical study.^2^

Gut microbiota is considered one of the key factors regulating host health. Gut microbiota can participate in the regulation of multiple metabolic pathways of the host, (1) forming metabolic interactions and signal transduction between the host and microbial flora and (2) physiologically connecting the immune-inflammatory axis of the gut, liver, muscle, and brain. It is also related to some gastrointestinal disorders, such as irritable bowel syndrome, chronic liver diseases, psychiatric disorders, metabolic diseases, hematologic diseases, and others. Fecal microbiota transplantation (FMT) has been applied to treat the aforementioned diseases. We suspected that POIS symptoms would be improved by regulating the gut microbiota.

## Case report

A patient with POIS visited our center eagerly seeking treatment with FMT. Here we report the first case of washed microbiota transplantation (WMT) for treating POIS.

In August 2022, a 20-year-old man came to our center who identified the first time of discomfort after ejaculation in January 2017. He immediately had a series of symptoms after masturbation and spermatorrhea, including extreme fatigue, dizziness, unresponsiveness, memory loss, apathy, lack of interest, irritability, runny nose, sneezing, excessive sweating, dryness, chills, headache, itchy eyes, palpitations, inattention, nervousness, loss, dyslalia, incoherent speech, and poor sleep quality. These symptoms would persist for 7 to 10 days and occur again after sexual activity. They had a negative effect on the patient’s study, daily, and social life and resulted in stopping sexual activity. Considering the 5 preliminary criteria proposed by Waldinger et al,^3^ the patient was diagnosed with POIS.

The patient visited several hospital in China from September 2017 to August 2021. Levels of progesterone, testosterone, estradiol, follicle-stimulating hormone, luteinizing hormone, prostate-specific antigen, and the thyroid gland were within normal limits. Results from the skin prick test and intracutaneous test with autologous semen were positive. B-type ultrasonography and brain magnetic resonance imaging showed the left varicocele and a microadenoma in the right pterygoid of the pituitary gland. Results of electrocardiography, brain electrical activity mapping, and paranasal sinus computerized tomography were almost normal.

Some scales were filled on September 13, 2021. The Symptom Checklist 90 (SCL-90) suggested that the patient had some recent physical and psychological discomfort. Scores on the Hamilton Anxiety Scale and Hamilton Depression Scale indicated that mild depression and mild anxiety were the main manifestations. The patient was diagnosed with mild depression and atypical bipolar disorder. The patient had taken many drugs from 2017 to 2022: fluvoxamine maleate, paroxetine, levocetirizine, loratadine, lithium carbonate sustained-release tablets, quetiapine fumarate tablets, sodium valproate, tamsulosin, and Chinese patent medicine. Although those would relieve some of the symptoms, the patient experienced side effects from these drugs. The patient also had undergone auto-semen desensitization treatment at the allergy department of the Second Affiliated Hospital of Harbin Medical University, but he thought that it did not work well.

Therefore, the patient was eager to find other effective treatments for POIS. The patient learned on the forum that the treatment of FMT would improve the symptoms of POIS. So he came to our department in August 2022.

This study was conducted and approved by the Ethics Committee (No. 2023-22) in accordance with the Declaration of Helsinki at the First Affiliated Hospital of Guangdong Pharmaceutical University, Guangzhou, China. The patient provided his written informed consent to participate in this study.

## Methods

At admission, the patient was well developed with normal secondary sexual characteristics and no obvious abnormalities on physical examination. Routine laboratory test results (blood, urine, stool, electrolytes, blood glucose, renal/liver function) and hormone levels (free T4, thyroid-stimulating hormone, cortisol, testosterone) were normal. Prolactin was 1186.85 mIU/L. After informed consent and the exclusion of immunosuppressive agents, decompensated liver cirrhosis, advanced AIDS, recent bone marrow transplantation, or other severe immunodeficiency, the patient received WMT treatment for 3 courses.

WMT is similar to traditional FMT in principle, but the difference between WMT and traditional FMT is that (1) a fecal bacteria intelligent separation system is used to obtain fecal bacteria and (2) repeated washing reduces harmful substances in fecal bacteria, such as increasing types and amount of viruses and metabolites with proinflammatory effects, such as leukotriene B4, corticosterone, and prostaglandin G2. WMT has been added as a safety measure. It has good safety, quality control, and effectiveness against diseases with floral disorders.^4^ The WMT procedure was in line with the “Nanjing Consensus on Methodology of Washed Microbiota Transplantation”*.*^5^ Our center implemented the standard “3 and 3 courses of treatment” of WMT, which means that there are 4 courses of treatment in total for a duration of 5 months. In the first 3 months, 1 course of WMT was carried out every month, and bacterial solution was injected into the ileocecal part each course, every day, for 3 consecutive days. Three months after the third course, the patient returned to consolidate treatment for 1 course. The patient received 3 courses of WMT.

To assess the severity of POIS and the efficacy of WMT, we developed a self-rating scale of POIS symptoms, referring to the symptoms for criterion 1 proposed by Waldinger et al in 2011.^3^ This scale reflects how the patient actually felt over the past month. Briefly, this included a sensation of a flu-like state, extreme fatigue or exhaustion, weakness of musculature, feverishness or perspiration, mood disturbances or irritability, memory difficulties, concentration problems, incoherent speech, congestion or watery nose, and itching eyes. According to the frequency of these symptoms after ejaculation, *no* is 0 points, *sometimes* is 1, *often* is 2, and *always* is 3. The Self-rating Anxiety Scale (SAS), Self-rating Depression Scale (SDS), SCL-90, and self-rating scale of POIS symptoms were completed before and 1 month after WMT.

## Results

After completing the first course of WMT, the patient felt that his sleep quality and digestive symptoms improved. Six days after the second course of WMT, the patient thought that the symptoms were 60% to 70% less severe than before WMT overall. Finally, 5 days after the third course of WMT, he thought that the symptoms were 30% to 40% less severe than the second course of WMT overall.

Table 1 shows the effects of WMT on the patient’s POIS symptoms. The results showed that after WMT, the symptoms of a flu-like state, extreme fatigue or exhaustion, weakness of musculature, and congestion or watery nose improved. Mood disturbances or irritability and memory difficulties were consistent with the baseline. Incoherent speech and itching eyes did not appear with the whole WMT.

**Table 1:** Clinical efficacy on postorgasmic illness syndrome during treatment of WMT.

	**Sensation of flu-like state**	**Extreme fatigue or exhaustion**	**Weakness of musculature**	**Feverishness or perspiration**	**Mood disturbances or irritability**	**Memory difficulties**	**Concentration problems**	**Incoherent speech**	**Nose congestion or watery nose**	**Itching eyes**	**Total**
Baseline	3	3	1	1	2	1	2	0	3	0	16
After WMT											
First	3	3	1	1	2	1	2	0	3	0	16
Second	1	1	0	1	1	1	1	0	2	0	8
Third	1	2	0	1	2	1	1	0	1	0	9

Abbreviation: WMT, washed microbiota transplantation.

Scale scores changed before and after WMT: SAS (before, 45; after first WMT, 42.5; after second, 37.5; after third, 45); SDS (63.75, 58.75, 47.5, 50), and SCL-90 (143, 140, 109, 149). Scores of the SAS, SDS, and SCL-90 all decreased after the first and second courses of WMT. Although the scores of SDS after the third course of WMT is higher than that after the second course, it is still lower than the baseline.

**Figure 1:**
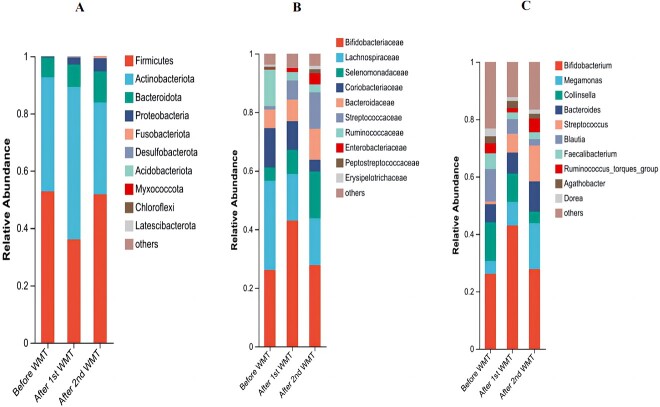
Gut microbiota composition in the patient before and after washed microbiota transplantation.

We analyzed gut microbiota composition in the patient before and after WMT. At the phylum level (Figure 1A), the relative abundance of Bacteroidota and Proteobacteria increased after WMT. At the family level (Figure 1B), the relative abundance of Selenomonadaceae and Streptococcaceae increased after WMT; however, the relative abundance of Lachnospiraceae, Coriobacteriaceae, and Bacteroidaceae decreased after WMT. At the genus level (Figure 1C), the relative abundance of *Bacteroides* (beneficial bacteria in most cases) and *Megamonas* (opportunistic pathogens) increased after WMT. The relative abundance of the following decreased after WMT: *Collinsella* (opportunistic pathogens in some cases and increased in the rectum of infertile men), *Blautia* (increased in the gut of people with major depressive disorder and negative correlation with sleep quality), *Faecalibacterium* (beneficial bacteria), *Fusicatenibacter* (rich in lung adenocarcinoma and positively associated with Sjogren’s syndrome), and *Dorea* (rich in autism spectrum disorder).

## Discussion

POIS, a rare disease that affects men’s health, was first described by Waldinger and Schweitzer in 2002 in their case reports.^1^ Symptoms of POIS occur rapidly after ejaculation and last for 4 to 7 days. However, the pathophysiology of POIS remains unclear. With the increase of diagnosis and treatment, many hypotheses on the pathophysiology of POIS have been proposed.

A substantial amount of evidence suggests that there is a relationship between stress/anxiety response and sexual dysfunction.^6^ Hu et al reported that gut microbiota transplantation from “healthy” Sprague-Dawley rats to fawn-hooded rats could suppress the aggravation of depression symptoms in the recipients through gut microbe–induced modulation of host immune and metabolic activity.^7^ Studies showed that in major depressive disorder, *Anaerostipes*, *Blautia*, *Clostridium*, *Klebsiella*, *Lachnospiraceae incretae sedis*, *Parabacteroides*, *Parasutterella*, *Phascolarctobacterium*, and *Streptococcus* levels were higher, and *Bifidobacterium*, *Dialister*, *Escherichia/Shigella*, *Faecalibacterium*, and *Ruminococcus* levels were lower.^8^ Our patient was in a state of moderate depression before WMT, which improved after WMT as the POIS symptoms improved. We suspect that depression may be associated with POIS symptoms.

Li et al reported that at the genus level, the relative abundance of *Enterococcus*, *Corynebacterium*, *Aerococcus*, and *Facklamia* (opportunistic pathogens in most cases) increased, and that of *Allobaculum*, *Bifidobacterium*, *Eubacterium*, and *Anaerotruncus* (beneficial bacteria) decreased in a group with type 2 diabetes and erectile dysfunction as compared with the second week after establishment of the type 2 diabetes model.^9^ Zhang et al reported that mice receiving A10-FMT (gut microbiota from alginate oligosaccharide, 10 mg/kg) had an increase in *Bacteroidales*, *Bifidobacteria*, *Sphingomonadales*, and *Campylobacterales.*^10^

In our case, at the genus level, the relative abundance of *Bacteroides* and *Megamonas* overall increased within a period of 3 courses of WMT, and the relative abundance of *Collinsella*, *Blautia*, *Faecalibacterium*, *Fusicatenibacter*, and *Dorea* decreased overall. Within 1 month after completing the first course of WMT, the patient felt that his sleep quality and digestive symptoms improved. Six days after the second course of WMT, the patient thought that the symptoms were 60% to 70% less severe than before WMT overall. Finally, 5 days after the third course of WMT, he thought that the symptoms were 30% to 40% less severe than the second course of WMT overall. So we speculate that the symptoms of POIS may be related to the characteristics of gut microbiota in patients.

## Conclusion

WMT may be an effective and safe choice for the treatment of patients with POIS. However, trials with larger sample sizes are needed to confirm the efficacy of this treatment.

## References

[1] Waldinger MD , Schweitzer DH. Postorgasmic illness syndrome: two cases. J Sex Marital Ther. 2002;28(3):251–255. 10.1080/009262302760328280 11995603

[2] Zizzo J , Sávio LF, Ramasamy R, Lima TFN. Postorgasmic illness syndrome: an update. Eur Urol Focus. 2023;9(1):22–24. 10.1016/j.euf.2022.09.016 36283946

[3] Waldinger MD , Meinardi MMHM, Zwinderman AH, Schweitzer DH. Postorgasmic illness syndrome (POIS) in 45 Dutch Caucasian males: clinical characteristics and evidence for an immunogenic pathogenesis (part 1). J Sex Med. 2011;8(4):1164–1170. 10.1111/j.1743-6109.2010.02166.x 21241453

[4] Zhang T , Lu G, Zhao Z, et al. Washed microbiota transplantation vs manual fecal microbiota transplantation: clinical findings, animal studies and in vitro screening. Protein Cell. 2020;11(4):251–266. 10.1007/s13238-019-00684-8 31919742 PMC7093410

[5] Shi Q . Nanjing consensus on methodology of washed microbiota transplantation. Chin Med J. 2020;133(19):2330–2332. 10.1097/CM9.0000000000000954 32701590 PMC7546843

[6] Tekin A , Meriç C, Sağbilge E. The relationship between childhood sexual/physical abuse and sexual dysfunction in patients with social anxiety disorder. Nord J Psychiatry. 2016;70(2):88–92. 10.3109/08039488.2015.1053097 26110606

[7] Hu B , das P, Lv X, et al. Effects of “healthy” fecal microbiota transplantation against the deterioration of depression in fawn-hooded rats. mSystems. 2022;7(3):e0021822. 10.1128/msystems.00218-22 35481347 PMC9239139

[8] Cheung SG , Goldenthal AR, Uhlemann AC, Mann JJ, Miller JM, Sublette ME. Systematic review of gut microbiota and major depression. Front Psychiatry. 2019;10:34. 10.3389/fpsyt.2019.00034 30804820 PMC6378305

[9] Li H , Qi T, Huang ZS, et al. Relationship between gut microbiota and type 2 diabetic erectile dysfunction in Sprague-Dawley rats. J Huazhong Univ Sci Technolog Med Sci. 2017;37(4):523–530. 10.1007/s11596-017-1767-z 28786059

[10] Zhang C , Xiong B, Chen L, et al. Rescue of male fertility following faecal microbiota transplantation from alginate oligosaccharide-dosed mice. Gut. 2021;70(11):2213–2215. 10.1136/gutjnl-2020-323593 33443023 PMC8515102

